# Exploration of Olfaction and ChiPSO in Pediatric Cystic Fibrosis

**DOI:** 10.3390/jcm14082583

**Published:** 2025-04-09

**Authors:** Tiana M. Saak, Jeremy P. Tervo, Brandon M. Moore, Alicia S. Wang, Emily DiMango, Hossein Sadeghi, David A. Gudis, Jonathan B. Overdevest

**Affiliations:** 1 Columbia University Vagelos College of Physicians and Surgeons, New York, NY 10032, USA; 2 Division of Pulmonary, Allergy, and Critical Care Medicine, New York Presbyterian, Columbia University Irving Medical Center, New York, NY 10032, USA; 3 Division of Pediatric Pulmonology, New York Presbyterian, Columbia University Irving Medical Center, New York, NY 10032, USA; 4 Department of Otolaryngology-Head and Neck Surgery, New York Presbyterian, Columbia University Irving Medical Center, New York, NY 10032, USA

**Keywords:** cystic fibrosis, children, olfaction, ChiPSO, olfactory importance, quality of life

## Abstract

**Background/Objectives**: Olfactory dysfunction (OD) is a common symptom among people with cystic fibrosis (PwCF) and contributes to environmental safety concerns, nutritional challenges, and an overall diminished quality of life. OD is perceived to progress along the lifespan in PwCF, often due to worsening sinonasal disease. Among children with cystic fibrosis (CwCF), OD is poorly characterized as limited resources and tolerance contribute to challenges in psychophysical olfactory evaluation among pediatric populations. The Children’s Personal Significance of Olfaction (ChiPSO) questionnaire was recently proposed as a tool to assess olfaction and the importance of olfactory stimulation among children. This pilot study aimed to evaluate the utility of ChiPSO among a cohort of ethnically diverse CwCF. **Methods**: Individuals aged 7–17 with physician-diagnosed CF were asked to complete questionnaires, including ChiPSO and the brief questionnaire on olfactory dysfunction (bQOD-NS), prior to undergoing psychophysical olfactory evaluation with the U-Sniff Identification test. Potential associations between questionnaires and olfactory performance, pulmonary function, and demographic characteristics were evaluated using Pearson and Spearman correlations, independent-sample *t*-tests, Wilcoxon rank sum tests, and multiple linear regression. **Results**: U-Sniff Identification score positively correlated with the overall ChiPSO total score [*r*(13) = 0.640, *p* = 0.010] and its environmental subdomain score [*r*(13) = 0.774, *p* < 0.001], though not with the food subdomain [*r*(13) = 0.450, *p* = 0.093], the social subdomain [*r*(13) = 0.343, *p* = 0.2], or bQOD-NS score [*r*(11) = −0.125, *p* = 0.7]. Hispanic ethnicity is associated with ChiPSO (*p* = 0.041). **Conclusions**: In this preliminary study, olfactory importance increases with olfactory function among an ethnically diverse sample of CwCF, with a preferential influence of olfactory function on personal importance of environmental olfactory information. While these results should be interpreted with limitations imposed by the pilot nature of our sample size, our pilot data highlights associations with early adolescent development of importance of olfaction that can be disrupted in the setting of progressive disease among CwCF.

## 1. Introduction

Cystic fibrosis (CF) is a genetic disorder caused by a mutation in the gene encoding the cystic fibrosis transmembrane conductance regulator (CFTR), leading to dehydrated, thickened mucous. As a result, patients often develop chronic pulmonary and gastrointestinal complications including infection, inflammation, nutritional deficiencies, and progressive loss of lung function [1,2]. Olfactory dysfunction (OD) is a common secondary feature of CF [3,4], likely due to chronic rhinosinusitis [5,6], olfactory cleft inflammation [4], and excessively viscous mucus that may impair odorant transmission to the olfactory cleft [7]. Most people with cystic fibrosis (PwCF) experience conductive olfactory impairment, with the prevalence among adults reaching 90% in some studies [4,8,9]. While OD is also a feature among children with CF (CwCF) [10,11], there is less known about olfaction and the prevalence of OD among pediatric populations [10,12,13]. Other common causes of OD in children result from impediments to normal nasal airflow including acute upper respiratory infections, chronic rhinosinusitis, severe allergic rhinitis, and adenoid hypertrophy, while more rare neurologic causes of OD in children include congenital anosmia, traumatic brain injury, and post-infectious etiologies [14,15]. The lack of standardized olfactory data in pediatric CF, and childhood OD more broadly, is attributable in part to the difficulty of psychophysical olfactory assessment in pediatric populations [16]. PwCF may also underreport sinonasal symptoms based on subjective measures due to the presence of symptoms from an early age that may result in reduced awareness of an asymptomatic state [17].

OD poses significant health concerns through reduced awareness of environmental dangers [18], diminished nutritional status [19,20,21], development of depression and loneliness [22,23], increased risk for cognitive impairment and neurodegenerative diseases [24], and diminished overall quality of life (QoL) [25,26,27]. In addition to conductive orthonasal olfactory dysfunction, diminished taste [28] and retronasal olfaction [29] in PwCF further exacerbate QoL and nutritional disruptions. Therefore, nutritional awareness is especially concerning in CwCF, a group at risk of growth and development issues and who require strict prescriptive diets [30,31]. OD may contribute to nutritional status in CwCF independently of pancreatic insufficiency and metabolic rate by altering appetite, enjoyment of food, and eating behaviors [11,27,32]. Furthermore, OD in childhood, whether acquired or congenital, may result in structural brain changes and neurodevelopment consequences [33]. Childhood OD is associated with the development of schizophrenia [34] and autism spectrum disorder [35,36], and OD is a well-known biomarker for Alzheimer’s Disease and Parkinson’s Disease later in life [24]. Thus, early detection and monitoring of OD among CwCF is critical to mitigate these potential downstream adverse effects.

Given the health burden posed by OD, there is interest in developing novel olfactory evaluation methods, with emerging models using olfactory-stimulated functional near-infrared spectroscopy [37], fMRI evaluation [38], and self-administered psychophysical olfactory assessments among mature adolescents and adults [39,40]. The Children’s Personal Significance of Olfaction (ChiPSO) questionnaire [41] was recently developed to offer a supplemental method for olfactory assessment that gathers perceptual insights from children and adolescents in how they interact with smells. Ratings using the ChiPSO instrument are significantly associated with psychophysical olfactory assessment in normative children and adolescents and provide insight into demographic features that may impact olfactory significance among children [41]. Additionally, ChiPSO quantifies the personal importance of olfactory information in three subdomains: environment, social, and food. As a new tool, evaluation of ChiPSO in broad pediatric population is only beginning [42,43] and no study has evaluated its utility in CwCF. The correlation of the ChiPSO questionnaire with psychophysical performance is unique, as many studies suggest no correlation between self-reported metrics of olfactory capabilities and psychophysical test performance in adult OD populations [44,45,46,47,48]. Given the difficulty of traditional olfactory assessment in pediatric populations and the increased burden of OD and OD-related sequelae among CwCF, ChiPSO may offer a well-tolerated, child-centric approach to olfactory assessment and insight into olfactory importance in pediatric CF, enabling clinicians to identify and address sequelae of OD. In this pilot phase of a prospective study, we sought to evaluate the utility of ChiPSO in an ethnically diverse cohort of CwCF.

## 2. Materials and Methods

Following screening of our clinical population of CwCF, 15 individuals were enrolled in the pilot phase of this study. Participants were recruited and evaluated in accordance with the proposed protocols (AAAU6106; 24/01/2025) approved by the Columbia University Irving Medical Center Institutional Review Board. A convenience sample was obtained by contacting patients from our panel of patients cared for within our pediatric Cystic Fibrosis Center. Families of CwCF who were scheduled for clinical appointments within six months of the start of our study were preferentially recruited to participate on the day of their regularly scheduled appointment to reduce financial and logistical burden. Informed consent and assent were retrieved from parents/legal guardians and interested participants, respectively, prior to participation in the study.

To participate in the study, the following inclusion criteria were applied: (1) age range of 7–17 years old and (2) physician-confirmed diagnosis of CF. A diagnosis of CF requires both clinical presentation of the disease and evidence of CFTR dysfunction, which can be confirmed by sweat chloride testing results ≥ 60 mmol/L, known CF-causing CFTR mutations, and/or CFTR dysfunction by CFTR physiologic testing [49]. Exclusion criteria included any potential acute process impacting sinonasal function at the time of enrollment: (1) experiencing an acute CF exacerbation (as defined by their pulmonologist or need for IV antibiotics) or (2) active upper respiratory tract infection. Additionally, participants were excluded if they were diagnosed with any rhinologic or neurologic disease that is known to impact olfactory function.

Participants provided basic demographic information, while clinical data, such as CF genotype and CFTR modulator therapy status, was obtained via chart review. FEV_1_ and FVC data was obtained via chart review, as well, from the most recent pulmonary function test each participant had completed. In-person testing was administered by a trained member of the research staff on the Columbia University Irving Medical Center campus in New York between 1 July 2023 and 31 July 2024, and the data collection instruments were selected specifically because of their adaptation for use in pediatric populations.

### 2.1. Study Questionnaires

Participants completed several questionnaires, including the Children’s Personal Significance of Olfaction (ChiPSO) questionnaire [41] (Table S1), a 15-item scale designed to assess the importance of olfactory information in three categories, food, social, and environmental, and the Brief Questionnaire of Olfactory Disorders—Negative Statements (bQOD-NS) [50] (Table S2), an olfactory-specific quality-of-life measure, with assistance from parent(s)/guardian(s) as necessary. The ChiPSO questionnaire includes self-rated personal statements evaluating their degree of agreement with each statement. Points are assigned for each of the 15 items as follows: 4: “I totally agree”; 3: “I mostly agree”; 2: “I mostly disagree”; 1 “I totally disagree”. Possible scores range from 15 to 60, with higher scores conveying a stronger sense of olfactory information importance. Subscores are calculated in each category by summing points assigned to the items as follows: social (2, 5, 6, 13, 15); environment (3, 6, 9, 11, 14); and food (1, 4, 7, 10, 12). Possible subscores range from 5 to 20.

Similarly, the bQOD-NS is a 7-item questionnaire comprising self-statements with points assigned for each item based on the degree of agreement (3: “Totally agree”; 2: “Partially agree”; 1: “Partially disagree”; 0: “Totally disagree”), with possible scores ranging from 0 to 21. Higher scores indicate a worse smell-related quality of life.

### 2.2. Olfactory Evaluation

Participants then underwent assessment of their psychophysical olfactory function using the Universal Sniff (U-Sniff) test (Burghart Messtechnik GmbH, Holm, Germany) [51], a 12-item variant of the Sniffin’ Sticks identification test that is validated for use in children <18 years old. This method employs the use of felt-tipped pens containing odorants dissolved in propylene glycol. Each odorant-filled pen was placed 2 cm in front of the participants’ nostrils for 3 s. Participants were asked to identify each odor out of 4 possible multiple-choice options. The target odorants are designed to be common, such that participants would be expected to encounter them in their everyday life (apple, banana, butter, coffee, cut grass, fish, flower, lemon, onion, orange, peach, and strawberry).

Score on the U-Sniff test is determined by the number of correctly identified odors out of 12 possible points. The Burghart Kids Identification Test Manual [52,53] contains score cutoffs by age range to determine normosmia versus reduced olfactory function, but this assessment is not sufficient to determine normosmia. For children ages 6–8, normosmia corresponds to a score ≥ 7; for ages 9–11, score ≥ 9 indicates normosmia, and adolescents 12–17 are considered normosmic with scores ≥ 10. Any score below the age-specific cutoff was considered hyposmia in the context of this study.

### 2.3. Statistical Analysis

IBM SPSS Statistics for macOS, version 29.0 (IBM Corp., Armonk, NY, USA), was used for all statistical analyses. Questionnaire and olfactory outcome variables for the entire study population and each categorical group (age, sex, and ethnicity) were assessed for normality using Shapiro–Wilk tests and Q-Q plots. ChiPSO, U-Sniff, and pulmonary function data for the overall study population and within each categorical group studied including age, sex, and ethnicity were found to be normally distributed around a mean. bQOD-NS data was not found to be normally distributed. Thus, all data was reported as mean (SD) except for bQOD-NS data, for which medians (IQR) are required.

Associations between ChiPSO and bQOD-NS questionnaires and olfactory performance, pulmonary function, and demographic characteristics were evaluated using Pearson and Spearman correlations, independent-sample *t*-tests, and Wilcoxon rank sum tests. Statistical significance was determined at *p* values < 0.05. All variables, except bQOD-NS, were found to be normally distributed. Therefore, Pearson correlations and independent-sample *t*-tests were sufficient for all analyses, except for those including bQOD-NS, where Spearman correlations and Wilcoxon rank sum tests were performed instead. Lastly, multiple linear regression of olfactory performance, pulmonary function, and demographic characteristics versus ChiPSO total score and ChiPSO environment subdomain score was performed to allow for controlling of age and sex.

## 3. Results

### 3.1. Study Demographics and Cystic Fibrosis History

ChiPSO, psychophysical olfactory assessment, and demographic surveys were completed for 15 children, of which 9 (60%) were female and 6 (40%) were male (Table 1). The mean (SD) age of participants was 12.73 (2.98), and eight (53%) participants identified as Hispanic or Latino/a. Nine (60%) participants had at least one ∆F508 allele on genetic testing, though six (40%) of participants had another recognized CF genotype. Two (13%) participants underwent endoscopic sinus surgery prior to participation in the study. Mean (SD) pulmonary function, as assessed by FEV_1_/FVC, was 87.52 (10.09). Mean (SD) BMI among study participants was 19.25 (3.54). Most participants (80%) were actively being treated with Elexacaftor/Tezacaftor/Ivacaftor (ETI) therapy. Among those being treated with modulator therapy, the mean (SD) time on the treatment was 25.83 (4.80) months.

### 3.2. Olfactory and Quality-of-Life Assessments

The mean (SD) U-Sniff Identification score was 9.40 (1.12) among the study population (Table S3). When adjusting for the age-specific score cutoffs, six (40%) participants were deemed to have OD. The mean (SD) ChiPSO score was 43.53 (8.24), with social, environment, and food subdomain mean (SD) scores of 13.53 (4.42), 15.00 (3.04), and 15.00 (3.12), respectively. Median (IQR) bQOD-NS score was 0 (0, 3).

**Figure 1:**
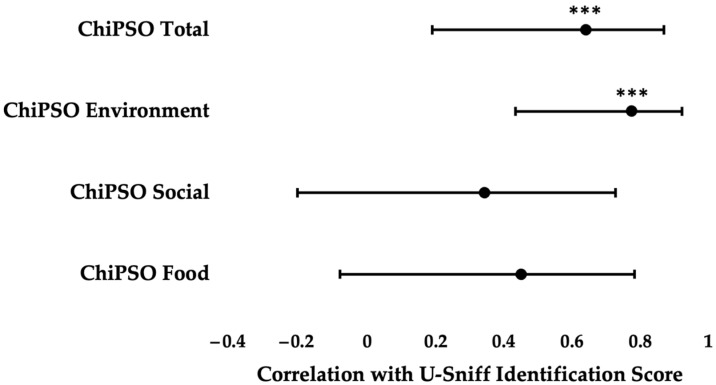
Pearson correlation coefficients and 95% confidence intervals of ChiPSO scores with U-Sniff Identification score among children with CF. *** *p* < 0.05.

Intact olfaction was associated with a stronger sense of olfactory information importance, especially for environment olfactory information. U-Sniff Identification score strongly correlated with both overall ChiPSO total score [*r*(13) = 0.640, *p* = 0.010] and the ChiPSO environment subdomain score [*r*(13) = 0.774, *p* < 0.001] (Figure 1 and Table S4). Both relationships persisted when controlling for age and sex ([coef. = 3.7, *p* = 0.043] (Table 2a) [coef. = 2.2, *p* = 0.003] (Table 2b), respectively). However, olfactory ability did not correlate with either of the other two ChiPSO subdomain scores of food [*r*(13) = 0.450, *p* = 0.093] or social [*r*(13) = 0.343, *p* = 0.2]. Further, there were no significant associations between olfaction and olfactory-related quality of life as measured by bQOD-NS [*r*(11) = −0.125, *p* = 0.7] (Table S4).

### 3.3. Impact of Pulmonary Function on Olfactory Importance and Quality-of-Life

We found no significant association between pulmonary function as evaluated by the FEV_1_/FVC score [*r*(13) = 0.290, *p* = 0.4] and ChiPSO or with bQOD-NS score [*r*(11) = 0.048, *p* = 0.9] (Table S5). When controlling for covariates of age and sex, neither the relationship between FEV_1_/FVC and ChiPSO total score [coef. = 0.14, *p* = 0.7] nor the relationship between FEV_1_/FVC and ChiPSO environment subdomain score [coef. = 0.11, *p* = 0.4] was found to be statistically significant (Table 2).

### 3.4. Demographic Considerations of Olfactory Importance and Quality-of-Life

**Figure 2:**
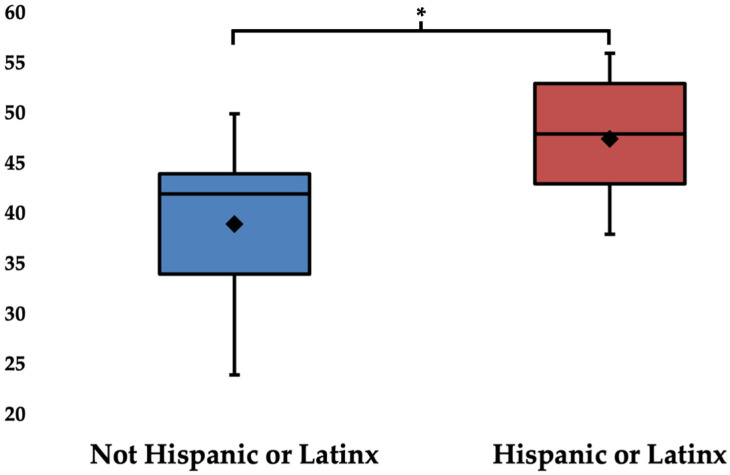
Total ChiPSO scores by self-reported ethnicity. ◊ mean value; * *p* < 0.05.

Hispanic participants reported a higher importance of olfaction with a mean (SD) ChiPSO score of 47.60 (6.00) as compared to 39.00 (8.47) in their non-Hispanic counterparts (*p* = 0.041) (Figure 2 and Table S6). This relationship persisted, and was slightly strengthened, when controlling for covariates of age and sex [coef. = 9.0, *p* = 0.034] (Table 2a). However, ethnicity was not associated with ChiPSO environment subdomain score [coef. = 1.3, *p* = 0.5] (Table 2b). ChiPSO total score and ChiPSO environment subdomain score were not significantly impacted by age or sex (Table 2 and Table S6). Further, age, sex, and self-reported ethnicity were not found to significantly associate with bQOD-NS score (Table S6).

## 4. Discussion

This pilot prospective study aimed to evaluate the utility of the ChiPSO olfactory-specific questionnaire as a measure to characterize pediatric olfactory function among CwCF. We assessed the relationship between overall olfactory importance and olfactory function using standardized psychophysical assessment, as well as with validated smell-related quality-of-life questionnaires. Additionally, we analyzed demographic associations with overall and subdomains of olfactory importance. Despite the limited sample size of this pilot study, our data suggest a preliminary correlation between both overall ChiPSO score and the environment subdomain score with olfactory function, as measured by U-Sniff Identification assessment. We also identified greater olfactory importance among children who identified as being ethnically Hispanic, though given the small sample size and lack of correction for multiple comparisons, this association should be cautiously interpreted.

Scoring convention for ChiPSO differs from bQOD-NS in that a higher ChiPSO score indicates a greater emphasis on importance of olfaction, while a higher bQOD-NS score indicates a more substantial quality of life impairment due to underlying OD. Among CwCF, the median ChiPSO score was comparable to the findings reported by Lohrer et al. [41] in their initial report of ChiPSO among a normative pediatric population. Notably, the bQOD-NS appears to have limited ability to stratify olfactory-specific quality of life among CwCF, mirroring prior studies demonstrating that across all ages of PwCF, there appears to be lower perceived impairments for olfactory-specific quality of life compared to patients with other forms of OD [6]. A component of these findings may be that children and adolescents have less agency over certain items represented on the bQOD-NS, such as going to restaurants to eat. Additionally, the chronicity and early onset of olfactory symptoms in PwCF may cause reduced awareness of normosmia and therefore a shift in patients’ concept of baseline quality of life. Taken together, children with OD may have difficulty in accurately characterizing olfactory-specific QoL, where this instrument appears to have limited stratification capacity in our CwCF population. Other reports of quality of life in the general population of PwCF have identified impairments related to olfactory function [27], with the olfactory-related negative QoL burden imparting significant psychological distress [54] and just 50% of adults with OD reporting overall satisfaction with their life [55]. Due to the known QoL disturbances among adults and OD prevalence of approximately 20% in the general population [56,57], further investigation may be warranted in this area, particularly as quality of life relates to olfactory importance among CwCF and pediatric populations more broadly.

Our finding that U-Sniff Identification score correlates with overall ChiPSO score is in line with the initial report of ChiPSO among a broader pediatric population studied by Lohrer et al. [41], where intact olfactory ability preliminarily corresponds to a greater sense of olfactory importance that was also observed in our cohort of CwCF. In the normative pediatric population [41], no correlation was found between odor identification and ChiPSO score among children aged 6–11, while a positive correlation was found in adolescents aged 12–17, suggesting that olfactory function has a greater contribution to olfactory importance with age. This further highlights a need for early emphasis on interventions that retain functional olfactory abilities among CwCF to sustain a sense of olfactory importance as they progress into adolescence and adulthood.

The relationship between U-Sniff Identification score and ChiPSO underscores the potential utility of ChiPSO as a low-impact way to evaluate olfactory function in children. While a deeper understanding of the strength of this association will require larger sample sizes of CwCF, our findings within a diverse cohort highlight the potential of ChiPSO to stratify olfactory function among CwCF using a simple, well-tolerated questionnaire. In addition to the overall ChiPSO score, the environment subdomain strongly correlated with psychophysical olfactory function among CwCF, which differs from the association between the U-Sniff score and the ChiPSO social subdomain in the normative pediatric population studied by Lohrer et al. [41]. Although differences in ChiPSO subdomain scores may reflect the limited power in the current study, future investigation of how olfaction relates to food and nutrition is warranted as many CwCF require high-calorie, high-fat diets to counteract pancreatic insufficiency. Even though different preferences for food exist among CwCF that may not entirely reflect chemosensory function [13], there are likely shared experiences with a broader pediatric population, where ChiPSO is negatively associated with vegetable acceptance [43].

An additional unique aspect of this study was the ethnically diverse population of CwCF, where half of the participants identified as Hispanic and Latino/a. Non-White and non-Caucasian individuals are not well-represented in CF research, and while there is an increasing appreciation for a more multi-ethnic and racially diverse community of PwCF [58,59,60], disparate and often worse health outcomes are observed in these underrepresented groups. There is also genotypic diversity among ethnic groups in PwCF, which may contribute to differences in phenotype and response to modulator therapy [61]. In the present study, Hispanic participants had significantly higher ChiPSO scores than non-Hispanic participants, conveying a greater sense of olfactory importance. The origin of this association is not clear, especially as there have been limited reports of the ChiPSO to date. Importantly, this association should be especially interpreted with caution, given the small sample size and lack of correction for multiple comparisons. While further expansion of this study will be necessary to provide validation of this association, these preliminary results are notable given the underrepresentation of Hispanic individuals in CF research.

The original ChiPSO still lacks formal validation and reliability studies against other measures of olfaction, though the method has been modified, evaluated, and validated in normative Chinese [42] and Swedish [43] children. While there have been studies that evaluate racial disparities of OD [62,63], there have been no studies that include an evaluation of how ethnic or cultural factors may influence ChiPSO in children. The experience of food, environmental, and social odors are deeply rooted in cultural norms and expression, and cultural differences in language surrounding olfaction may also contribute to variation in perceived odor importance [64,65,66]. Therefore, further validation of psychophysical olfactory assessments and subjective assessments of olfaction, such as ChiPSO, is required among CwCF with diverse racial and ethnic backgrounds to ensure proper clinical application.

While there is not yet a distinct path for prevention or improvement of OD among PwCF, treatment of rhinosinusitis is a priority in CF management [67]. Current therapeutic options targeting symptoms of chronic rhinosinusitis in CF include topical steroid treatment, topical antibiotic delivery, surgical intervention, and increasing reliance on CFTR modulator therapies, though there is not yet strong agreement for how to utilize all of these treatment options amongst PwCF [68,69,70]. There is hope that early CFTR modulator therapy initiation may help improve retention of olfaction among PwCF, though additional studies are needed due to discrepancies between self-reported olfactory function and psychophysical measures of olfaction among those who have initiated treatment [6,71,72]. Additionally, OD seems to persist among adults despite long-term use of modulator therapies [73], emphasizing the importance of studying longitudinal olfactory outcomes in children starting modulator therapy to prevent effects of chronic rhinosinusitis that may impart irreversible OD. Toward this goal, future longitudinal studies investigating modulator therapies in CwCF may incorporate the ChiPSO as a screening measure to trend olfactory performance.

Regarding olfactory recovery and retention efforts among CwCF, olfactory training has shown promise in childhood olfactory deficits following traumatic brain injury [74] and may help mitigate primary headache pain in children [75]. Studies evaluating olfactory training in CwCF are needed to assess other potential low-risk treatment options. Lastly, olfactory-related QoL and mental health concerns were particularly highlighted during the COVID-19 pandemic [54,76,77], and further research is necessary regarding QoL, and mental health metrics validated in CwCF and other pediatric OD are needed to apply these findings to children.

A strength of this pilot study is the utilization of tools specifically designed to assess pediatric psychophysical olfactory performance and subjective olfactory importance among a population of CwCF. Further, our cohort of CwCF represents diversity of age, sex, and ethnicity among study participants. Our study also has limitations, the most significant being a limited sample size given the pilot nature of this prospective investigation that curbs the generalizability of our conclusions and challenges our ability to perform sufficiently powered subgroup analyses. However, this study provides a framework to pursue further investigation into the utility of ChiPSO in CwCF, particularly as a compliment and possible surrogate to using the U-Sniff Identification test, which itself is limited in that it solely assesses the identification domain of olfaction. Further investigation examining other domains of smell such as threshold or discrimination in children is warranted. ChiPSO as an assessment tool is limited in its applicability in very young children, who may have difficulty with self-reported questionnaires. As a result, ChiPSO has yet to be studied in children under the age of six. Additionally, the study was limited due to potential selection bias as recruitment occurred from a convenience sample of children presenting for regular treatment of CF. These children may not be representative of the larger pediatric CF population in terms of disease management or social support factors.

## 5. Conclusions

Our pilot study presents data demonstrating the utility of a new measure, ChiPSO, in quantifying olfactory importance among a diverse cohort of CwCF. We demonstrate that psychophysical assessment of olfactory function is associated with an increased overall ChiPSO score, consistent with normative data from a broad pediatric population. In contrast to normative data where ChiPSO social subdomain score correlates with olfactory function, we show that the environment subdomain score correlates with psychophysical olfactory function in CwCF. Lastly, our preliminary findings suggest that Hispanic ethnicity may be associated with an increased sense of olfactory importance among CwCF. Overall, ChiPSO has utility in the early identification of potential OD among CwCF and merits use in further investigation with large, diverse pediatric CF populations to better understand sustained olfactory importance.

## Figures and Tables

**Table 1:** Demographics and descriptive statistics.

	**N = 15 ^1^**
**Age**	12.73 (2.98)
**Sex**	
Female	9 (60%)
Male	6 (40%)
**Ethnicity**	
Hispanic or Latino/a	8 (53%)
Non-Hispanic or Latino/a	7 (47%)
**History of endoscopic sinus surgery**	2 (13%)
**Cystic fibrosis genotype**	
Other	6 (40%)
∆F508/minimal function	3 (20%)
∆F508/residual function	1 (7%)
∆F508/∆F508	5 (33%)
**FEV_1_/FVC (%)**	87.52 (10.09)
**BMI**	19.25 (3.54)
**Active CFTR modulator usage**	
Elexacaftor/Tezacaftor/Ivacaftor (ETI)	12 (80%)
Not eligible or prior failure	3 (20%)
**Time on ETI (months)**	25.83 (4.80)

^1^ Mean (SD); n (%).

**Table 2:** (a) Multiple linear regression of olfactory function, pulmonary function, and demographic measures versus ChiPSO total score. (b) Multiple linear regression of olfactory function, pulmonary function, and demographic measures versus ChiPSO environment score.

**(a)**			
**Metric**	**Coef. ^1^**	**CI**	***p*-Value**
**U-Sniff Identification Score**	3.7	−0.19, 7.6	0.043
**FEV_1_/FVC (%)**	0.14	−0.58, 0.87	0.7
**Ethnicity**			
Hispanic versus non-Hispanic	9.0	0.80, 17	0.034
Age *			
Adolescents versus children	2.9	−7.5, 13	0.6
Sex *			
Female versus male	6.7	−2.6, 16	0.014
**(b)**			
**U-Sniff Identification Score**	2.2	0.91, 3.4	0.003
**FEV_1_/FVC (%)**	0.11	−0.15, 0.36	0.4
**Ethnicity**			
Hispanic versus non-Hispanic	1.3	−2.5, 5.1	0.5
Age *			
Adolescents versus children	1.5	−2.5, 5.4	0.4
Sex *			
Female versus male	2.0	−1.6, 5.5	0.3

^1^ Multiple linear regression; covariates: age and sex. * For age regression, sex was covariate; for sex regression, age was covariate.

## Data Availability

The original contributions presented in this study are included in the article. Further inquiries can be directed to the corresponding author.

## Supplementary Materials

The following supporting information can be downloaded at https://www.mdpi.com/article/10.3390/jcm14082583/s1: Table S1: ChiPSO questionnaire; Table S2: bQOD-NS questionnaire; Table S3: Questionnaire and olfactory test scores; Table S4: Correlation of psychophysical olfactory performance with olfactory importance and QoL metrics; Table S5: Correlation of pulmonary function with olfactory importance and QoL metrics; Table S6: Association of demographic characteristics with olfactory importance and QoL metrics.

## Abbreviations

The following abbreviations are used in this manuscript:

BMI	body mass index
bQOD-NS	Brief Questionnaire of Olfactory Disorder—Negative Statements
CF	cystic fibrosis
CFTR	cystic fibrosis transmembrane conductance regulator
ChiPSO	Children’s Personal Significance of Olfaction
CwCF	children with cystic fibrosis
ETI	Elexacaftor/Tezacaftor/Ivacaftor
FEV_1_	forced expiratory volume in 1 s
FVC	forced vital capacity
OD	olfactory dysfunction
PwCF	people with cystic fibrosis
QoL	quality of life
U-Sniff	Universal Sniff Identification test
