# Supplementary material for: Exploration of Olfaction and ChiPSO in Pediatric Cystic Fibrosis
Source: J Clin Med. 2025 Apr 9;14(8):2583. doi: 10.3390/jcm14082583 (PMC12027488; doi:10.3390/jcm14082583)
Supplement: Supplementary file 1 [file jcm-14-02583-s001.zip › JCM_TableS1_finalproof.pdf]

Table S1. ChiPSO questionnaire.

		Totally agree	Mostly agree	Mostly disagree	Totally disagree
1	I sniff on food before eating				
2	It happens that I smell my clothes				
3	When I walk on the street, I normally smell something of the surrounding				
4	When I don't like the smell of a food I don't eat it				
5	I find that my parents smell of something				
6	It would bother me if there were no odors anymore				
7	If there is a dish on the table that I don't know I would smell it before eating				
8	It happens that I smell parts of my body				
9	When I smell an odor around me, I try to guess what it is				
10	Smelling nasty odors while eating usually makes me lose my appetite				
11	I normally try to find out where an odor comes from				
12	The smell of a food plays a role in the decision whether I like it or not				
13	I smell my clothes to check whether it has to be washed				
14	I like to smell the odors around me when I walk in nature				
15	I smell myself to check whether I have a bad odor				

* social subscore includes items 2, 5, 8, 13, and 15; environment subscore includes items 3, 6, 9, 11, and 14; food subscore includes items 1, 4, 7, 10, and 12.

** 4 points are awarded for "I totally agree"; 3 for "I mostly agree"; 2 for "I mostly disagree"; 1 for "I totally disagree".
