# Supplementary material for: Exploration of Olfaction and ChiPSO in Pediatric Cystic Fibrosis
Source: J Clin Med. 2025 Apr 9;14(8):2583. doi: 10.3390/jcm14082583 (PMC12027488; doi:10.3390/jcm14082583)
Supplement: Supplementary file 1 [file jcm-14-02583-s001.zip › JCM_tableS2_finalproof.pdf]

Table S2. bQOD-NS questionnaire.

		Totally agree	Partially agree	Partially disagree	Totally disagree
1	The changes in my sense of smell make me feel isolated				
2	Because of the changes in my sense of smell I have problems with taking part in activities of daily life				
3	The changes in my sense of smell make me feel angry				
4	Because of the changes in my sense of smell, I go to restaurants less often than I used to				
5	Because of the changes in my sense of smell, I eat less than I used to or more than I used to				
6	Because of the changes in my sense of smell, I try harder to relax				
7	I am worried that I will never get used to the changes in my sense of smell				

* Here, 3 points are awarded for "Totally agree"; 2 points for "Partially agree"; 1 point for "Partially disagree"; 0 points for "Totally disagree".
