# Supplementary material for: Exploration of Olfaction and ChiPSO in Pediatric Cystic Fibrosis
Source: J Clin Med. 2025 Apr 9;14(8):2583. doi: 10.3390/jcm14082583 (PMC12027488; doi:10.3390/jcm14082583)
Supplement: Supplementary file 1 [file jcm-14-02583-s001.zip › JCM_TableS3_finalproof.pdf]

Table S3. Questionnaire and olfactory test scores.

Metric	N = 15[1]
**ChiPSO Total Score**	43.53 (8.24)
Social Subdomain Score	13.53 (4.42)
Environment Subdomain Score	15.00 (3.04)
Food Subdomain Score	15.00 (3.12)
**Brief QOD-NS Score**	0 (0, 3)
No response	2 (13%)
**U-Sniff Identification Score**	9.40 (1.12)
**Categorical OD**	6 (40%)

[1] Mean (SD); median (IQR); n (%)
