# Supplementary material for: Exploration of Olfaction and ChiPSO in Pediatric Cystic Fibrosis
Source: J Clin Med. 2025 Apr 9;14(8):2583. doi: 10.3390/jcm14082583 (PMC12027488; doi:10.3390/jcm14082583)
Supplement: Supplementary file 1 [file jcm-14-02583-s001.zip › JCM_TableS4_finalproof.pdf]

Table S4. Correlation of psychophysical olfactory performance with olfactory importance and QoL metrics.

Questionnaire	U-Sniff Identification[1]	p-value
**ChiPSO Total Score**	0.640	0.010[2]
Social Subdomain Score	0.343	0.2[2]
Environment Subdomain Score	0.774	< 0.001[2]
Food Subdomain Score	0.450	0.093[2]
**Brief QOD-NS Score**	-0.125	0.7[3]

[1] Correlation coefficient.

[2] Pearson correlation.

[3] Spearman correlation.
