# Supplementary material for: Exploration of Olfaction and ChiPSO in Pediatric Cystic Fibrosis
Source: J Clin Med. 2025 Apr 9;14(8):2583. doi: 10.3390/jcm14082583 (PMC12027488; doi:10.3390/jcm14082583)
Supplement: Supplementary file 1 [file jcm-14-02583-s001.zip › JCM_TableS5_finalproof.pdf]

Table S5. Correlation of pulmonary function with olfactory importance and QoL metrics.

Questionnaire	FEV_1/FVC (%)[1]	p-value
ChiPSO Total Score	0.290	0.4[2]
Brief QOD-NS Score	0.048	0.9[3]

[1] Correlation coefficient.

[2] Pearson correlation.

[3] Spearman correlation.
