# Supplementary material for: Exploration of Olfaction and ChiPSO in Pediatric Cystic Fibrosis
Source: J Clin Med. 2025 Apr 9;14(8):2583. doi: 10.3390/jcm14082583 (PMC12027488; doi:10.3390/jcm14082583)
Supplement: Supplementary file 1 [file jcm-14-02583-s001.zip › JCM_TableS6_finalproof.pdf]

Table S6. Association of demographic characteristics with olfactory importance and QoL metrics.

Demographic characteristic	**ChiPSO,** N=15[1]	**p-value**[2]	**QOD-NS,** N=13[3]	**p-value**[4]
**Age**				
Adolescents (N=11)	43.91 (6.44)	0.8	0 (0, 7.0)	0.5
Children (N=4)	42.50 (13.30)		0 (0, 2.25)	
**Sex**				
Male (N=6)	39.50 (8.50)	0.13	0 (0, 11.5)	0.7
Female (N=9)	46.22 (7.31)		0 (0, 2.75)	
**Ethnicity**				
Hispanic (N=8)	47.60 (6.00)	0.041	3.0 (0, 8.0)	0.14
Non-Hispanic (N=7)	39.00 (8.47)		0 (0, 0.5)	

[1] Mean (SD).

[2] Independent-sample t-test.

[3] Median (IQR).

[4] Wilcoxon rank sum test.
